# A CD8^+^ T cell-associated immune gene panel for prediction of the prognosis and immunotherapeutic effect of melanoma

**DOI:** 10.3389/fimmu.2022.1039565

**Published:** 2022-10-20

**Authors:** Shanwen Sun, Zhengke Zhi, Yang Su, Jingxian Sun, Qianjun Li

**Affiliations:** ^1^ Department of Medical Oncology, The Affiliated Huai’an Hospital of Xuzhou Medical University and The Second People’s Hospital of Huai’an, Huaian, China; ^2^ Department of Pediatric Surgery, Children’s Hospital of Nanjing Medical University, Nanjing, China; ^3^ Jiangsu Center for the Collaboration and Innovation of Cancer Biotherapy, Cancer Institute, Xuzhou Medical University, Xuzhou, China; ^4^ Hypertension Research Institute of Geriatric Hospital of Nanjing Medical University, Jiangsu Province Official Hospital, Nanjing, China; ^5^ Department of Gastroenterology, The Affiliated Huaian No.1 People’s Hospital of Nanjing Medical University, Huaian, China

**Keywords:** CD8+ T cell, immune gene, prognosis, immunotherapeutic effect, melanoma

## Abstract

**Background:**

Skin cutaneous melanoma (SKCM) is the most frequently encountered tumor of the skin. Immunotherapy has opened a new horizon in melanoma treatment. We aimed to construct a CD8^+^ T cell-associated immune gene prognostic model (CDIGPM) for SKCM and unravel the immunologic features and the benefits of immunotherapy in CDIGPM-defined SKCM groups.

**Method:**

Single-cell SKCM transcriptomes were utilized in conjunction with immune genes for the screening of CD8^+^ T cell-associated immune genes (CDIGs) for succeeding assessment. Thereafter, through protein-protein interaction (PPI) networks analysis, univariate COX analysis, and multivariate Cox analysis, six genes (*MX1*, *RSAD2*, *IRF2*, *GBP2*, *IFITM1*, and *OAS2*) were identified to construct a CDIGPM. We detected cell proliferation of SKCM cells transfected with *IRF2* siRNA. Then, we analyzed the immunologic features and the benefits of immunotherapy in CDIGPM-defined groups.

**Results:**

The overall survival (OS) was much better in low-CDIGPM group versus high CDIGPM group in TCGA dataset and GSE65904 dataset. On the whole, the results unfolded that a low CDIGPM showed relevance to immune response-correlated pathways, high expressions of *CTLA4* and *PD-L1*, a high infiltration rate of CD8^+^ T cells, and more benefits from immunotherapy.

**Conclusion:**

CDIGPM is an good model to predict the prognosis, the potential immune escape from immunotherapy for SKCM, and define immunologic and molecular features.

## Introduction

Pigment cells originate from the neural crest locate in the epidermis, in which they primarily serve as protectors for keratinocytes against UV-elicited DNA injury (1, 2). Skin cutaneous melanoma (SKCM), developing from vicious transformation of melanocytes, turns out to be the fatal form of skin cancer (3). The proportion of new SKCM cases is mounting in spite of the decreasing proportions of most cancers. Moreover, SKCM causes about 72% of deaths in skin cancer on account of its strong potentials in metabolism and metastasis (4, 5).

Recently, immunotherapy has revolutionized the therapy for cancer. In particular, on the condition of the approval of the use of *CTLA-4*, *PD-L1*, and *PD-1*-specific immune checkpoint inhibitors, immunotherapy performs better than conventional therapies in lengthening the overall survival (OS) of cases of heterogeneous tumors (6–9). SKCM is a class of tumor displaying the most sensitive to immunotherapeutic methods (10, 11). According to the latest clinical study, after SKCM patients underwent nivolumab + ipilimumab combination therapy, the 5-year OS rate was 52% (12). In addition, it appears that T cells are promotors for immune responses and immunotherapy, among which CD8^+^ T cells occupy a dominant position (13, 14). It is worth noting that CD8+ T cells play an important role in the prognosis of melanoma. It has been reported that the oxidative phosphorylation CD8+ T cell subset is predictive of immunotherapy resistance in melanoma patients (15), and CD8+ T-cell infiltration could influence patient survival in cutaneous melanoma directly (16). Therefore, tumor-infiltrating CD8^+^ T cells-associated immune genes (CDIGs) are probably targets for the identification of SKCM patients with sensitivity to immunotherapy.

Investigating prognostic markers for SKCM is central to this study, which can be conducive to the prediction of traditional therapeutic outcomes and the suggestion of immunotherapeutic value. For a detailed assessment of CD8+ T cell-related genes (the differential genes in CD8+ T cells) in SKCM, we explored the single-cell RNA sequencing (scRNA-seq) dataset using the Tumor Immune Single-Cell Hub (TISCH). Here, we constructed the CDIGs prognostic model (CDIGPM). Furthermore, we described the immunological characteristics of CDIGPM defined groups. Finally, we detected the ability of CDIGPM to predict the prognosis and immunotherapeutic efficacy in SKCM patients. Our research show that CDIGPM is an encouraging prognostic model.

## Materials and methods

### Recognition of CDIGs in SKCM

The 708 differentially expressed genes (DEGs) in CD8^+^ T cells in SKCM (**Table S1**) were obtained from TISCH (GSE120575, http://tisch.comp-genomics.org/). As a scRNA-seq database concentrating on the tumor microenvironment (TME), TISCH enables the exploration of TME. The filtering of DEGs in CD8^+^ T cells was based on the threshold of P < 0.05 and | log2 FC| ≥ 0.5. Meanwhile, the updated immune genes were retrieved from ImmPort and InnateDB. Later, DEGs of CD8^+^ T cells and immune genes were intersected to obtain CDIGs (**Table S2**), which would be examined later in this study.

### Recognition of hub CDIGs

Through the online database STRING (https://string‐db.org/), we generated a PPI network of CDIGs. Then, 32 hub CDIGs were filtered by the number of adjacent nodes ≥ 30.

### Construction of the CDIGPM

From 32 hub CDIGs, using univariate and multivariate Cox regression analyses, six genes (*MX1*, *RSAD2*, *IRF2*, *GBP2*, *IFITM1*, and *OAS2*) were screened to construct a CDIGPM (**Table S3**). In the model, we calculated the CDIGPM score using the formula: CDIGPM = [Expression value × gene coefficient]. After that, we drawn Kaplan-Meier survival curves and conducted a log-rank test to explore the performance of the CDIGPM on the TCGA and GEO cohorts. Additionally, by application of univariate and multivariate Cox regression analyses, we clarified independent prognostic value of the CDIGPM.

### Cell proliferation detection

The proliferation of SKCM cells was detected by Cell Counting Kit-8 kit (Beyotime Biotechnology, Shanghai, China). Approximately 1×10^3^ cells were incubated in triplicate in 96-well plates. At 48h, the Cell Counting Kit -8 reagent (10μL) was added to each well and incubated at 37°C for 2h. Absorbance at 450 nm was used.

### Immunologic features and immunotherapeutic effect in the two CDIGPM groups

Limma package of R was used for differential expression analysis of all genes. ClusterProfiler package of R was used for gene set enrichment analysis (GSEA) on GO and KEGG gene sets. Genetic alteration data were downloaded from TCGA for gene mutation analysis. Correlation analyses were performed between CDIGPM and *CTLA4* and *CD274* expressions.

To determine immune features of SKCM samples in different CDIGPM subgroups, we used CIBERSORT (https://cibersort.stanford.edu/) to calculate the relative proportion of 22 types of immune cells. The relative proportions of 22 types of immune cells were then compared between the two CDIGPM subgroups, and the results were showed in a landscape map.

### Statistical analysis

The continuous variables were analyzed by *t*-test. The categorical data were analyzed by χ^2^ test. Kaplan-Meier survival analysis and the log-rank test were used for univariate survival analysis. Cox regression model was used for multivariate survival analysis. P value < 0.05 was considered to be significant differences.

## Results

### Hub CDIGs

**Figure 1:**
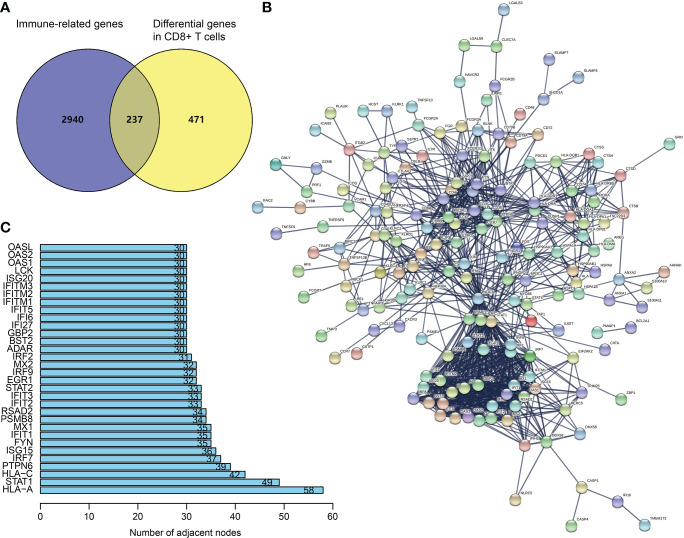
Recognition of hub CDIGs. **(A)** The overlapped genes of DEGs in CD8^+^ T cells in SKCM and IRGs. **(B)** The PPI network of CDIGs. **(C)** Hub CDIGs screened by the number of adjacent nodes ≥ 30. DEGs: differentially expressed genes, CDIGs: CD8^+^ T cells-associated immune genes.

From TISCH (http://tisch.comp-genomics.org/), we obtained 708 DEGs in CD8^+^ T cells of SKCM (**Table S1**). The filtering of DEGs in CD8^+^ T cells was based on the threshold of P < 0.05 and | log2 FC| ≥ 0.5. Meanwhile, the updated immune genes were retrieved from ImmPort and InnateDB. Later, DEGs in CD8^+^ T cells and immune genes were intersected to obtain CDIGs (**Figure 1A**; **Table S2**). Through PPI (STRING; https://cn.string-db.org), 32 hub CDIGs were screened (adjacent node count ≥ 30) (**Figures 1B, C**).

### CDIGPM

**Figure 2:**
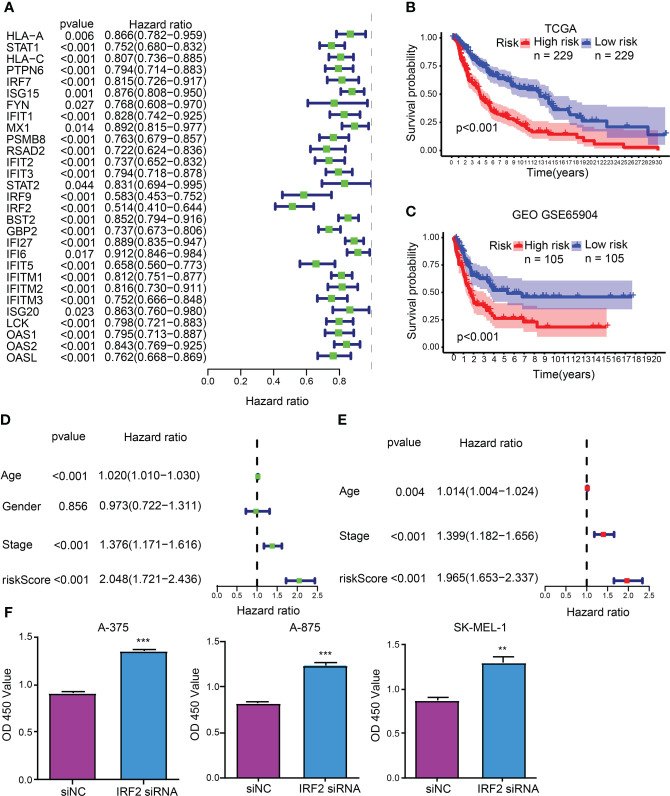
CDIGPM. **(A)** 29 hub CDIGs show remarkable relevance to OS according to univariate Cox regression analysis. **(B)** Kaplan-Meier survival analysis of high- and low-CDIGPM groups in the TCGA cohort. **(C)** Kaplan-Meier survival analysis of the high- and low-CDIGPM groups in the GEO cohort. **(D)** Univariate Cox analysis of clinical factors and the CDIGPM. **(E)** Multivariate Cox analysis of the factors significant in the univariate Cox analysis. **(F)** Cell proliferation of SKCM cells transfected with IRF2 siRNA or siNC. (**p < 0.01, ***p < 0.001). CDIGs: CD8^+^ T cells-associated immune genes, CDIGPM: CD8^+^ T cells-associated immune genes prognostic model, siNC: siRNA negative control.

As unveiled by the univariate Cox regression analysis, totaling 29 from 32 hub CDIGs (P < 0.05) were clearly connected with OS in TCGA cohort (**Figure 2A**; **Figure S1**). Then, the multivariate Cox regression analysis disclosed that six genes (*MX1*, *RSAD2*, *IRF2*, *GBP2*, *IFITM1*, and *OAS2*) were prognostic hallmarks, which were employed to build a CDIGPM (**Table S3**). In Cox model, the CDIGPM score of all samples were calculated using the formula: CDIGPM = [Expression value × gene coefficient] (**Table S3**). We then explored the expression of these genes in SKCM using GEPIA (http://gepia.cancer-pku.cn/). Based on TCGA and GTEx data, the expression levels of MX1, RSAD2, IRF2, GBP2, IFITM1, and OAS2 were showed in the **Figure S2**.

The performance of the CDIGPM in prognosis prediction was verified by the Kaplan-Meier survival curve and log-rank test on the TCGA and GEO cohorts. With the cutoff value of the median CDIGPM, samples fell into low-CDIGPM group and high CDIGPM group. It was disclosed that low-CDIGPM patients had better OS *vs.* high-CDIGPM patients (**Figures 2B, C**). We then evaluated the independent prognostic value of CDIGPM *via* univariate and multivariate Cox regression analyses. Results showed that score of CDIGPM, tumor stage and age (P < 0.05) were independent prognostic factors (**Figures 2D, E** **;** **Table S4**). Since the absolute value of *IRF2* coefficient is the largest (-0.419071422539304), we wonder whether it affects the prognosis by regulating the malignant behavior of tumor cells. Results showed that down-regulation of *IRF2* promoted SKCM cell proliferation (**Figure 2F**).

### Molecular features in high- and low-CDIGPM groups

**Figure 3:**
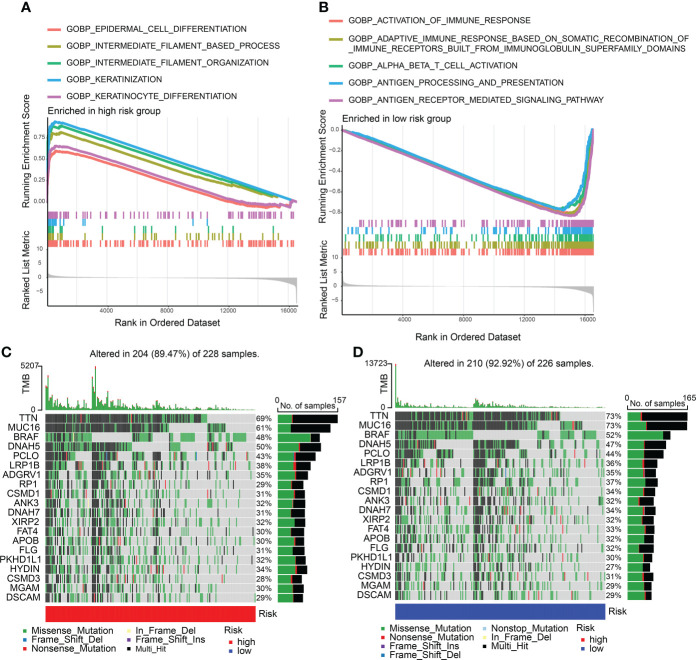
GSEA and mutation in high- and low-CDIGPM groups. **(A)** GO gene sets enriched in high-CDIGPM group. **(B)** GO gene sets enriched in low-CDIGPM group. **(C, D)** Significantly mutated genes in the mutated SKCM samples in high-CDIGPM group **(C)** and low-CDIGPM group **(D)**. Mutated genes (rows) are ordered by mutation rate; samples (columns) are arranged to emphasize mutual exclusivity among mutations. The right shows mutation percentage, and the top shows the overall number of mutations. The color-coding indicates the mutation type. CDIGPM: CD8^+^ T cells-associated immune genes prognostic model.

Enriched GO gene sets in the two CDIGPM groups were determined using GSEA. The GSEA plot illustrated top five pathways. The results uncovered the enrichment of epidermal cell differentiation, intermediate filament-based process, keratinization, intermediate filament organization, and keratinocyte differentiation in high-CDIGPM group (**Figure 3A**). Further, low-CDIGPM was connected immune responses related pathways (**Figure 3B**). Enriched KEGG gene sets in the two CDIGPM groups were showed in **Figure S3**.

Next, gene mutation analysis was performed and the gene mutation in low-CDIGPM score group and high-CDIGPM score group was showed in the **Figures 3C, D**.

**Figure 4:**
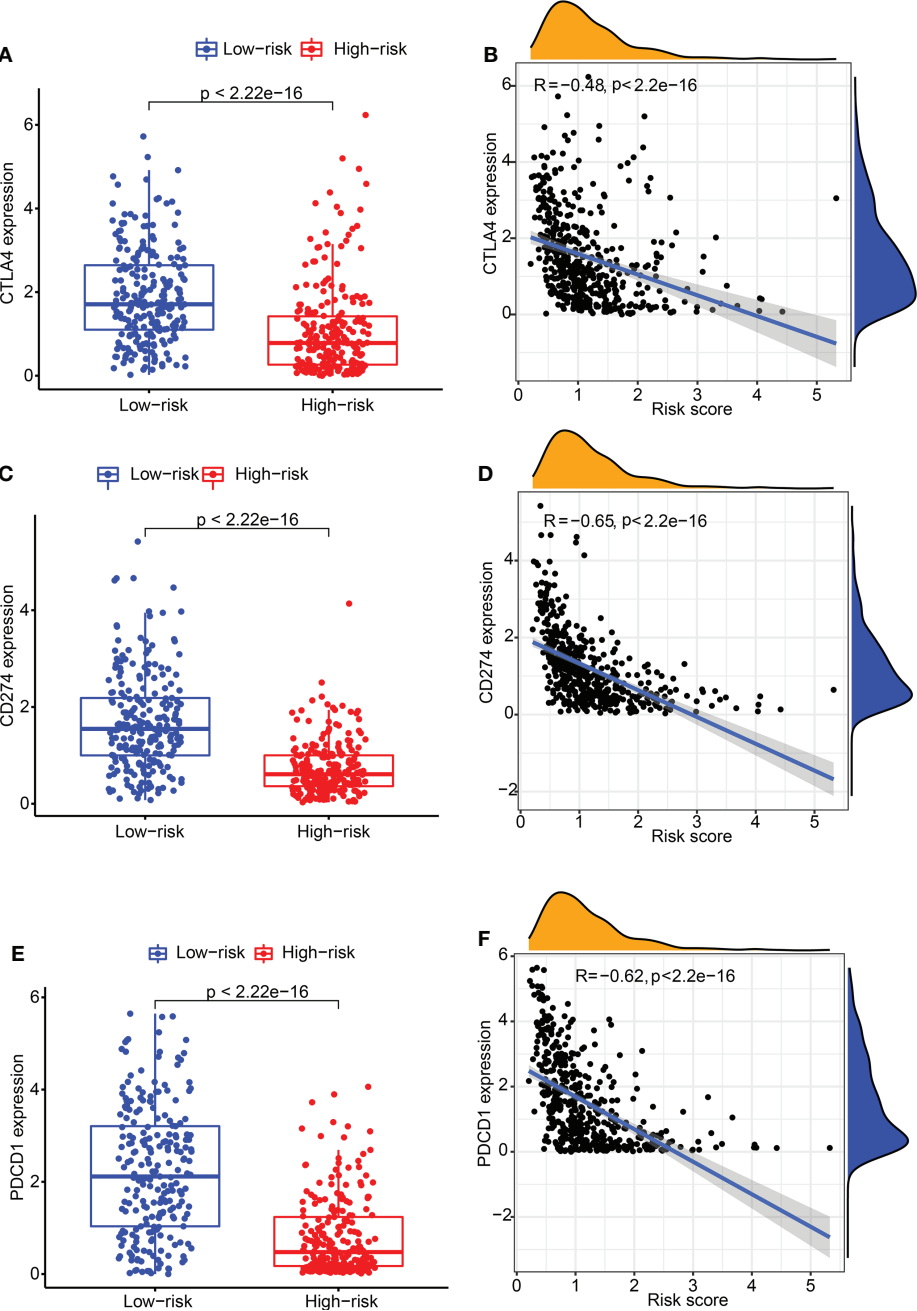
The expression of CTLA4 and CD274 in high- and low-CDIGPM groups. **(A)** *CTLA4* expression in high- and low-CDIGPM groups. **(B)** Correlation analysis between CDIGPM and *CTLA4* expression. **(C)** *CD274* expression in high- and low-CDIGPM groups. **(D)** Correlation analysis between CDIGPM and *CD274* expression. **(E)** *PDCD1* expression in high- and low-CDIGPM groups. **(F)** Correlation analysis between CDIGPM and *PDCD1* expression. CDIGPM: CD8^+^ T cells-associated immune genes prognostic model.

Subsequently, the relation between CDIGPM score and immune checkpoint genes were explored. It followed that the CDIGPM score displayed negative relevance to *CTLA4* expression (**Figures 4A, B**), *CD274* expression (**Figures 4C, D**), and *PDCD1* (*PD1*) expression (**Figures 4E, F**).

### Immunologic features of the two CDIGPM groups

**Figure 5:**
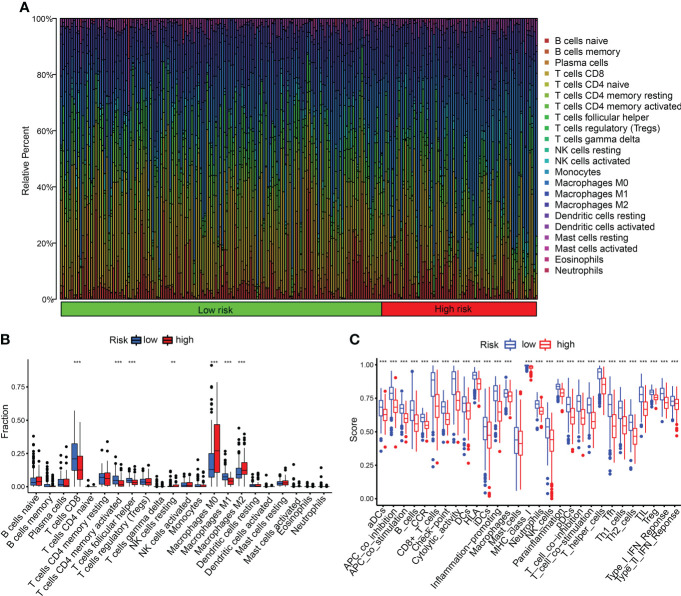
Immune characteristics in high- and low-CDIGPM groups. **(A, B)** The proportions of TME cells in high- and low-CDIGPM groups. **(C)** The molecular and immune-related function in high- and low-CDIGPM groups. (**p < 0.01, ***p < 0.001). CDIGPM: CD8^+^ T cells-associated immune genes prognostic model.

We detected the constituents of immune cells in the two CDIGPM groups. The results uncovered more activated memory CD4^+^ T cells, M1 macrophages (anti-tumor phenotype), and CD8^+^ T cells in low-CDIGPM group, but more M2 macrophages (pro-tumor phenotype) in high-CDIGPM group (**Figures 5A, B**). Then, we defined the immune and molecular function between the two groups by certain gene signatures. As a result, the immune and molecular function were more active in low-CDIGPM group (**Figure 5C**).

### Relationship between CDIGPM and clinical subtypes

**Figure 6:**
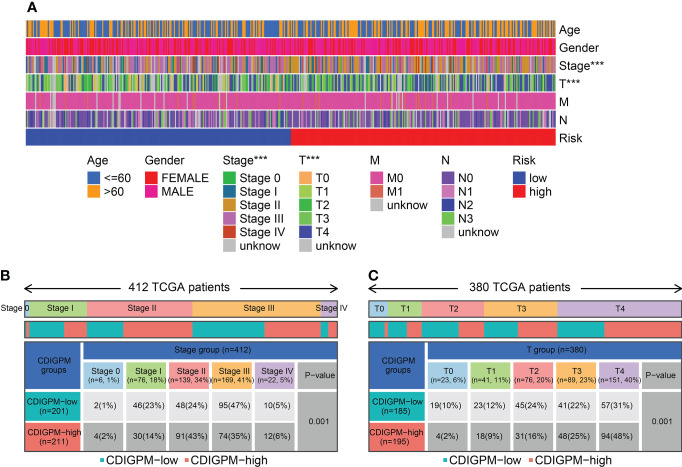
Relationship between CDIGPM and clinical subtypes. **(A)** The CDIGPM groups and clinical subtypes for SKCM patients in the TCGA cohort. Age, gender, tumor grade and TNM stage are shown as patient annotations. **(B)** Heat map showing the distribution of SKCM TNM stages (stage 0-IV) between high- and low-CDIGPM groups. **(C)** Heat map showing the distribution of SKCM grade (T0-4) between high- and low-CDIGPM groups. (***p < 0.001). CDIGPM: CD8^+^ T cells-associated immune genes prognostic model.

**Figure 6A** shows the clinical features in high- and low-CDIGPM groups. We could find from **Figures 6B, C** that CDIGPM was related to tumor stage and size (P = 0.001, χ^2^ test). Specifically, there were more Stage I samples and fewer Stage II samples in the low-CDIGPM group versus the high-CDIGPM group (P = 0.001). In **Figure 6C**, more T0-T2 samples were belonged to the low-CDIGPM group and more T3-T4 samples were belonged to the high-CDIGPM group (P = 0.001).

### Relationship between CDIGPM and immunotherapy

**Figure 7:**
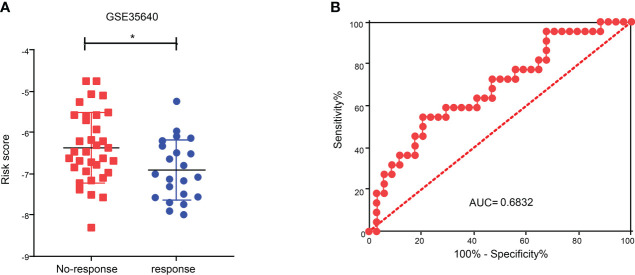
Relationship between CDIGPM and immunotherapy. **(A)** CDIGPM in patients who did not respond to immunotherapy is higher than it in patients who responded to immunotherapy. **(B)** ROC curve. (*p < 0.05). CDIGPM: CD8^+^ T cells-associated immune genes prognostic model.

To explore the role of CDIGPM in immunotherapeutic effect, we analyzed the expression profile in SKCM patients (GSE35640). The analysis yielded that the CDIGPM in SKCM patients who responded to immunotherapy was lower than it in SKCM patients who did not respond to immunotherapy (**Figure 7A**). Furthermore, receiver operating characteristic (ROC) analysis was performed to determine the performance of CDIGPM score in immunotherapeutic efficacy prediction (**Figure 7B**). Our findings mean that CDIGPM could predict whether there is a response to immunotherapy in SKCM patients. The graphical abstract of this research is displayed in the **Figure S4**.

## Discussion

Immunotherapy is the most promising therapy for several tumors, including melanoma. Its mechanism is to activate autologous immune responses by interfering with the tolerance of human cancer and re-inducing the tumor-resistant impacts on the TME (8, 17–19). Nevertheless, some patients couldn’t get satisfactory efficacies due to the complex mechanisms underlying tumor immunity (20). Melanoma treatment has recently made headway with the advent of immunotherapies (anti-CTLA4 and anti-PD-1 antibodies). Whereas sustained responses may be observed with anti-PD-1 antibody therapy, around 60% of patients still develop resistance (12, 21). Cancer cell phenotype plasticity (22, 23), tumor microenvironment (24), the expression of immune checkpoint genes (25) and other factors may be related to immune escape in melanoma. What’s more, the reported genomic and immune biomarkers are not accurate enough in evaluating therapeutic effects (26). Hence, though it is challenging, finding a better predictor is in sore need for the accurate assessment of clinical outcomes before immunotherapy.

T cells are tumor-resistant effector cells of paramount significance owing to their direct attacks on cancer cells. It has been recently presented that the outcome of immune checkpoint therapy (ICT) targeting T cells is promising in melanoma cases. The efficacy of ICT is only favorable in certain tumor cases, which appears to be affected by the extent of the activation or infiltration of immune cells, especially CD8^+^ T lymphocytes (16, 27, 28). From GSE120575, which was deposited in the TISCH, 708 DEGs of CD8^+^ T cell from SKCM were obtained. Then, the overlapped genes of immune genes and differential genes in CD8+ T cells were regarded as CDIGs. Through PPI, 32 hub CDIGs were screened for subsequent analysis. We screened 29 CDIGs pertaining to the prognosis of SKCM from these hub genes by univariate Cox analysis. By application of multivariate Cox regression analysis, we built the CDIGPM. Using TCGA and GEO arrays, the CDIGPM was proven to be an effective model for the prognosis of SKCM.

CDIGPM is composed of six genomes: *MX1*, *RSAD2*, *IRF2*, *GBP2*, *IFITM1*, and *OAS2*. *MX1* is an interferon-inducible dynamin GTPase that is essential for the suppression of replication of multifold viruses (29). It impedes the early stage in the replication cycle of discrepant viruses to exert inhibitory effect on these viruses (30). *RSAD2*, a gene stimulated by interferons, is engaged in congenital immunity and primarily accountable to antiviral responses. It is reported that knockdown of RSAD2 makes mature dendritic cells unable to stimulate the production of proinflammatory cytokines and T cell proliferation (31). Besides the participation in antiviral immune responses, *RSAD2* is also a potent driver for adaptive immune responses mediated by mature dendritic cells (31). *IRF2*, a constitutive transcription factor pertaining to cancer development, could exert anti-oncogenic activities by regulating tumor cell apoptosis, growth, and drug resistance (32, 33). *GBP2* is indispensable for the protective immunity against microorganisms (34). Moreover, up-regulation of *GBP2* expression corresponds to a better prognosis of breast cancer patients, and might participate in T-cell defense against breast cancer (35). *IFITM1*, belonging to the IFN-induced transmembrane protein family, exhibits high expressions in tumor tissues and cells, and it is an independent prognostic biomarker for patients suffering from tumors including gallbladder carcinoma, esophageal adenocarcinoma, colorectal cancer, and gastric cancer (36). According to a report, *OAS2* exists in the patients with malignant diseases (37). It appears that the high mRNA expression of OAS2 represents more favorable outcomes in breast cancer patients (38). In our CDIGPM, the coefficients of *MX1* and *OAS2* were positive numbers, while those of *RSAD2*, *IRF2*, *GBP2* and *IFITM1* were negative numbers. Therefore, CDIGPM is negatively correlated with *RSAD2*, *IRF2*, *GBP2* and *IFITM1* but positively correlated with *MX1* and *OAS2*. Since the absolute value of *IRF2* coefficient is the largest (-0.419071422539304), we wonder whether it affects the prognosis by regulating the malignant behavior of tumor cells. Results showed that down-regulation of *IRF2* promoted SKCM cell proliferation. It has been reported that IRF2 inhibits cell proliferation by inducing CLDN7 upregulation in oral squamous cell carcinoma (39), and inhibits cancer proliferation by promoting AMER-1 transcription in human gastric cancer (40). What’s means that CDIGPM might affects the prognosis of SKCM patients by regulating the malignant behavior of tumor cells. In summary, CDIGPM is a model that is related to the prognosis and tumor immunotherapy.

Then, we explored the correlation of CDIGPM with existing predictive markers including *CD274* (*PD-L1*) and *CTLA4* for immunotherapy. *CD274* ^+^ and *CTLA4* ^+^ tumors tend to respond better to ICT than negative tumors (41–43). Here, we found a negative correlation between CDIGPM score and *CTLA4* and *CD274*.

Exploring the TME might be helpful for finding new methods for the immunotherapy of SKCM. Between the two CDIGPM subgroups, there were differences in the activity of immune functions and the infiltration of certain immune cells. More CD8^+^ T cells, activated CD4^+^ memory T cells, and M1 macrophages were existed in low-CDIGPM group, while more M2 macrophages were existed in high-CDIGPM group. It has been reported that more infiltration of CD8^+^ T cells is related to a good prognosis in cancers (44–46). Activated M1 macrophages can trigger adaptive immune responses. M2 macrophages play an immunosuppressive role and exert a tumor growth-promoting effect (47, 48). All these mean that low-CDIGPM group has better tumor immunity potential, while high-CDIGPM group has immunosuppressive characteristics.

Aimed to explore its predictive value in cancer immunotherapy, we analyzed immunotherapy data GSE35640. We found that the CDIGPM score in patients who respond to immune therapy was lower than it in patients who did not respond to immune therapy. These results indicate that CDIGPM might be a prediction model for the effect of cancer immunotherapy.

Nevertheless, the study still has shortcomings. Most importantly, prospective studies are needed to further confirm the value of this prognostic model.

In total, CDIGPM is an encouraging prognostic model. It may help identify immunologic features and predict the prognosis of SKCM patients. Meanwhile, CDIGPM might have predictive value for immune escape.

## Data availability statement

The datasets presented in this study can be found in online repositories. The names of the repository/repositories and accession number(s) can be found below: https://www.ncbi.nlm.nih.gov/geo/, GSE120575.

## Author contributions

QL and YS made important contributions to the study conception and design. SS, JS and ZZ conducted data analysis and interpretation. All authors contributed to the article and approved the submitted version.

## Funding

This research was supported by Natural Science Research Plan of Huaian (No. HAB202022).

## Conflict of interest

The authors declare that the research was conducted in the absence of any commercial or financial relationships that could be construed as a potential conflict of interest.

The reviewer YX declared a shared affiliation with the authors, ZZ, JS, QL, to the handling editor at the time of the review.

## Publisher’s note

All claims expressed in this article are solely those of the authors and do not necessarily represent those of their affiliated organizations, or those of the publisher, the editors and the reviewers. Any product that may be evaluated in this article, or claim that may be made by its manufacturer, is not guaranteed or endorsed by the publisher.

## Supplementary material

Supplementary Figure 1 Kaplan-Meier curves of 29 hub CDIGs. Kaplan-Meier survival analysis of 29 hub CDIGs in the TCGA cohort. DEGs: differentially expressed genes.

Supplementary Figure 2 The expression levels of CDIGPM genes in SKCM. The expression levels of MX1, RSAD2, IRF2, GBP2, IFITM1, and OAS2 in SKCM were explored using GEPIA. Based on TCGA and GTEx data, the expression levels of MX1 and OAS2 are higher in SKCM cells. CDIGPM: CD8^+^ T cells-associated immune genes prognostic model.

Supplementary Figure 3 Enriched KEGG gene sets in high- and low-CDIGPM groups. **(A)** KEGG gene sets enriched in high-CDIGPM group. **(B)** KEGG gene sets enriched in low-CDIGPM group. CDIGPM: CD8^+^ T cells-associated immune genes prognostic model.

Supplementary Figure 4 Graphical Abstract.

## References

[1] Strashilov S Yordanov A. Aetiology and pathogenesis of cutaneous melanoma: Current concepts and advances. Int J Mol Sci (2021) 22(12):6395. doi: 10.3390/ijms22126395 34203771 PMC8232613

[2] Lopes F Sleiman MG Sebastian K Bogucka R Jacobs EA Adamson AS. UV Exposure and the risk of cutaneous melanoma in skin of color: A systematic review. JAMA Dermatol (2021) 157(2):213–9. doi: 10.1001/jamadermatol.2020.4616 33325988

[3] Gomez-Abenza E Ibanez-Molero S Garcia-Moreno D Fuentes I Zon LI Mione MC. Zebrafish modeling reveals that SPINT1 regulates the aggressiveness of skin cutaneous melanoma and its crosstalk with tumor immune microenvironment. J Exp Clin Cancer Res (2019) 38(1):405. doi: 10.1186/s13046-019-1389-3 31519199 PMC6743187

[4] Huang B Han W Sheng ZF Shen GL. Identification of immune-related biomarkers associated with tumorigenesis and prognosis in cutaneous melanoma patients. Cancer Cell Int (2020) 20:195. doi: 10.1186/s12935-020-01271-2 32508531 PMC7249670

[5] Guy GP Jr. Thomas CC Thompson T Watson M Massetti GM Richardson LC. Vital signs: melanoma incidence and mortality trends and projections - united states, 1982-2030. MMWR Morb Mortal Wkly Rep (2015) 64(21):591–6. PMC4584771 26042651

[6] Gu S Qian L Zhang Y Chen K Li Y Wang J. Significance of intratumoral infiltration of b cells in cancer immunotherapy: From a single cell perspective. Biochim Biophys Acta Rev Cancer (2021) 1876(2):188632. doi: 10.1016/j.bbcan.2021.188632 34626740

[7] Huppert LA Green MD Kim L Chow C Leyfman Y Daud AI. Tissue-specific tregs in cancer metastasis: opportunities for precision immunotherapy. Cell Mol Immunol (2022) 19(1):33–45. doi: 10.1038/s41423-021-00742-4 34417572 PMC8752797

[8] Zhu Y Zhu X Tang C Guan X Zhang W. Progress and challenges of immunotherapy in triple-negative breast cancer. Biochim Biophys Acta Rev Cancer (2021) 1876(2):188593. doi: 10.1016/j.bbcan.2021.188593 34280474

[9] Agarwal P Le DT Boland PM. Immunotherapy in colorectal cancer. Adv Cancer Res (2021) 151:137–96. doi: 10.1016/bs.acr.2021.03.002 34148613

[10] Fukuda K. Networks of CD8(+) T cell response activation in melanoma and vitiligo. Front Immunol (2022) 13:866703. doi: 10.3389/fimmu.2022.866703 35432377 PMC9011047

[11] Fujimura T. Stromal factors as a target for immunotherapy in melanoma and non-melanoma skin cancers. Int J Mol Sci (2022) 23(7):4044. doi: 10.3390/ijms23074044 35409404 PMC8999844

[12] Larkin J Chiarion-Sileni V Gonzalez R Grob JJ Rutkowski P Lao CD. Five-year survival with combined nivolumab and ipilimumab in advanced melanoma. N Engl J Med (2019) 381(16):1535–46. doi: 10.1056/NEJMoa1910836 31562797

[13] Hossain MA Liu G Dai B Si Y Yang Q Wazir J. Reinvigorating exhausted CD8(+) cytotoxic T lymphocytes in the tumor microenvironment and current strategies in cancer immunotherapy. Med Res Rev (2021) 41(1):156–201. doi: 10.1002/med.21727 32844499

[14] Pasetto A Lu YC. Single-cell TCR and transcriptome analysis: An indispensable tool for studying T-cell biology and cancer immunotherapy. Front Immunol (2021) 12:689091. doi: 10.3389/fimmu.2021.689091 34163487 PMC8215674

[15] Li C Phoon YP Karlinsey K Tian YF Thapaliya S Thongkum A. A high OXPHOS CD8 T cell subset is predictive of immunotherapy resistance in melanoma patients. J Exp Med (2022) 219(1):e20202084. doi: 10.1084/jem.20202084 34807232 PMC8611729

[16] Zhu G Su H Johnson CH Khan SA Kluger H Lu L. Intratumour microbiome associated with the infiltration of cytotoxic CD8+ T cells and patient survival in cutaneous melanoma. Eur J Cancer (2021) 151:25–34. doi: 10.1016/j.ejca.2021.03.053 33962358 PMC8184628

[17] Rawding PA Bu J Wang J Kim DW Drelich AJ Kim Y. Dendrimers for cancer immunotherapy: Avidity-based drug delivery vehicles for effective anti-tumor immune response. Wiley Interdiscip Rev Nanomed Nanobiotechnol (2022) 14(2):e1752. doi: 10.1002/wnan.1752 34414690 PMC9485970

[18] Lodewijk I Nunes SP Henrique R Jeronimo C Duenas M Paramio JM. Tackling tumor microenvironment through epigenetic tools to improve cancer immunotherapy. Clin Epigenet (2021) 13(1):63. doi: 10.1186/s13148-021-01046-0 PMC7992805 33761971

[19] Lizardo DY Kuang C Hao S Yu J Huang Y Zhang L. Immunotherapy efficacy on mismatch repair-deficient colorectal cancer: From bench to bedside. Biochim Biophys Acta Rev Cancer (2020) 1874(2):188447. doi: 10.1016/j.bbcan.2020.188447 33035640 PMC7886024

[20] Boone CE Wang L Gautam A Newton IG Steinmetz NF. Combining nanomedicine and immune checkpoint therapy for cancer immunotherapy. Wiley Interdiscip Rev Nanomed Nanobiotechnol (2022) 14(1):e1739. doi: 10.1002/wnan.1739 34296535 PMC8906799

[21] Wolchok JD Chiarion-Sileni V Gonzalez R Rutkowski P Grob JJ Cowey CL. Overall survival with combined nivolumab and ipilimumab in advanced melanoma. N Engl J Med (2017) 377(14):1345–56. doi: 10.1056/NEJMoa1709684 PMC5706778 28889792

[22] Benboubker V Boivin F Dalle S Caramel J. Cancer cell phenotype plasticity as a driver of immune escape in melanoma. Front Immunol (2022) 13:873116. doi: 10.3389/fimmu.2022.873116 35432344 PMC9012258

[23] Plaschka M Benboubker V Grimont M Berthet J Tonon L Lopez J. ZEB1 transcription factor promotes immune escape in melanoma. J Immunother Cancer (2022) 10(3):e003484. doi: 10.1136/jitc-2021-003484 35288462 PMC8921918

[24] Simiczyjew A Dratkiewicz E Mazurkiewicz J Zietek M Matkowski R Nowak D. The influence of tumor microenvironment on immune escape of melanoma. Int J Mol Sci (2020) 21(21):8359. doi: 10.3390/ijms21218359 PMC7664679 33171792

[25] Kakavand H Rawson RV Pupo GM Yang JYH Menzies AM Carlino MS. PD-L1 expression and immune escape in melanoma resistance to MAPK inhibitors. Clin Cancer Res (2017) 23(20):6054–61. doi: 10.1158/1078-0432.CCR-16-1688 28724663

[26] Hu-Lieskovan S Bhaumik S Dhodapkar K Grivel JJB Gupta S Hanks BA. SITC cancer immunotherapy resource document: a compass in the land of biomarker discovery. J Immunother Cancer (2020) 8(2):e000705. doi: 10.1136/jitc-2020-000705 33268350 PMC7713206

[27] Dreno B Khammari A Fortun A Vignard V Saiagh S Beauvais T. Phase I/II clinical trial of adoptive cell transfer of sorted specific T cells for metastatic melanoma patients. Cancer Immunol Immunother (2021) 70(10):3015–30. doi: 10.1007/s00262-021-02961-0 PMC8423703 34120214

[28] Leon-Letelier RA Castro-Medina DI Badillo-Godinez O Tepale-Segura A Huanosta-Murillo E Aguilar-Flores C. Induction of progenitor exhausted tissue-resident memory CD8(+) T cells upon salmonella typhi porins adjuvant immunization correlates with melanoma control and anti-PD-1 immunotherapy cooperation. Front Immunol (2020) 11:583382. doi: 10.3389/fimmu.2020.583382 33240271 PMC7682137

[29] Das BK Roy P Rout AK Sahoo DR Panda SP Pattanaik S. Molecular cloning, GTP recognition mechanism and tissue-specific expression profiling of myxovirus resistance (Mx) protein in labeo rohita (Hamilton) after poly I:C induction. Sci Rep (2019) 9(1):3956. doi: 10.1038/s41598-019-40323-0 30850653 PMC6408538

[30] Haller O Staeheli P Schwemmle M Kochs G. Mx GTPases: dynamin-like antiviral machines of innate immunity. Trends Microbiol (2015) 23(3):154–63. doi: 10.1016/j.tim.2014.12.003 25572883

[31] Jang JS Lee JH Jung NC Choi SY Park SY Yoo JY. Rsad2 is necessary for mouse dendritic cell maturation *via* the IRF7-mediated signaling pathway. Cell Death Dis (2018) 9(8):823. doi: 10.1038/s41419-018-0889-y 30068989 PMC6070531

[32] Guo Y Xu J Du Q Yan Y Geller DA. IRF2 regulates cellular survival and lenvatinib-sensitivity of hepatocellular carcinoma (HCC) through regulating beta-catenin. Transl Oncol (2021) 14(6):101059. doi: 10.1016/j.tranon.2021.101059 33735820 PMC7988337

[33] Liang C Zhang X Wang HM Liu XM Zhang XJ Zheng B. MicroRNA-18a-5p functions as an oncogene by directly targeting IRF2 in lung cancer. Cell Death Dis (2017) 8(5):e2764. doi: 10.1038/cddis.2017.145 28471447 PMC5520692

[34] Yu S Yu X Sun L Zheng Y Chen L Xu H. GBP2 enhances glioblastoma invasion through Stat3/fibronectin pathway. Oncogene (2020) 39(27):5042–55. doi: 10.1038/s41388-020-1348-7 32518375

[35] Godoy P Cadenas C Hellwig B Marchan R Stewart J Reif R. Interferon-inducible guanylate binding protein (GBP2) is associated with better prognosis in breast cancer and indicates an efficient T cell response. Breast Cancer (2014) 21(4):491–9. doi: 10.1007/s12282-012-0404-8 23001506

[36] Liang R Li X Zhu X. Deciphering the roles of IFITM1 in tumors. Mol Diagn Ther (2020) 24(4):433–41. doi: 10.1007/s40291-020-00469-4 32394410

[37] Gu X Boldrup L Coates PJ Fahraeus R Nylander E Loizou C. Epigenetic regulation of OAS2 shows disease-specific DNA methylation profiles at individual CpG sites. Sci Rep (2016) 6:32579. doi: 10.1038/srep32579 27572959 PMC5004144

[38] Zhang Y Yu C. Prognostic characterization of OAS1/OAS2/OAS3/OASL in breast cancer. BMC Cancer (2020) 20(1):575. doi: 10.1186/s12885-020-07034-6 32560641 PMC7304174

[39] Li X Yang W. IRF2-induced claudin-7 suppresses cell proliferation, invasion and migration of oral squamous cell carcinoma. Exp Ther Med (2022) 23(1):7. doi: 10.3892/etm.2021.10929 34815759 PMC8593875

[40] Chen YJ Luo SN Wu H Zhang NP Dong L Liu TT. IRF-2 inhibits cancer proliferation by promoting AMER-1 transcription in human gastric cancer. J Transl Med (2022) 20(1):68. doi: 10.1186/s12967-022-03275-0 35115027 PMC8812234

[41] Hansen AR Siu LL. PD-L1 testing in cancer: Challenges in companion diagnostic development. JAMA Oncol (2016) 2(1):15–6. doi: 10.1001/jamaoncol.2015.4685 26562503

[42] Oliva M Spreafico A Taberna M Alemany L Coburn B Mesia R. Immune biomarkers of response to immune-checkpoint inhibitors in head and neck squamous cell carcinoma. Ann Oncol (2019) 30(1):57–67. doi: 10.1093/annonc/mdy507 30462163 PMC6336003

[43] Pai CS Simons DM Lu X Evans M Wei J Wang YH. Tumor-conditional anti-CTLA4 uncouples antitumor efficacy from immunotherapy-related toxicity. J Clin Invest (2019) 129(1):349–63. doi: 10.1172/JCI123391 PMC6307943 30530991

[44] Bindea G Mlecnik B Tosolini M Kirilovsky A Waldner M Obenauf AC. Spatiotemporal dynamics of intratumoral immune cells reveal the immune landscape in human cancer. Immunity (2013) 39(4):782–95. doi: 10.1016/j.immuni.2013.10.003 24138885

[45] Fridman WH Zitvogel L Sautes-Fridman C Kroemer G. The immune contexture in cancer prognosis and treatment. Nat Rev Clin Oncol (2017) 14(12):717–34. doi: 10.1038/nrclinonc.2017.101 28741618

[46] Zhuang H Zhang C Hou B. FAM83H overexpression predicts worse prognosis and correlates with less CD8(+) T cells infiltration and ras-PI3K-Akt-mTOR signaling pathway in pancreatic cancer. Clin Transl Oncol (2020) 22(12):2244–52. doi: 10.1007/s12094-020-02365-z 32424701

[47] Yan K Wang Y Lu Y Yan Z. Coexpressed genes that promote the infiltration of M2 macrophages in melanoma can evaluate the prognosis and immunotherapy outcome. J Immunol Res (2021) 2021:6664791. doi: 10.1155/2021/6664791 33748290 PMC7959968

[48] Mantovani A Sozzani S Locati M Allavena P Sica A. Macrophage polarization: tumor-associated macrophages as a paradigm for polarized M2 mononuclear phagocytes. Trends Immunol (2002) 23(11):549–55. doi: 10.1016/s1471-4906(02)02302-5 12401408

