# Supplementary figures and images for: A CD8+ T cell-associated immune gene panel for prediction of the prognosis and immunotherapeutic effect of melanoma
Source: Front Immunol. 2022 Oct 20;13:1039565. doi: 10.3389/fimmu.2022.1039565 (PMC9633226; doi:10.3389/fimmu.2022.1039565)

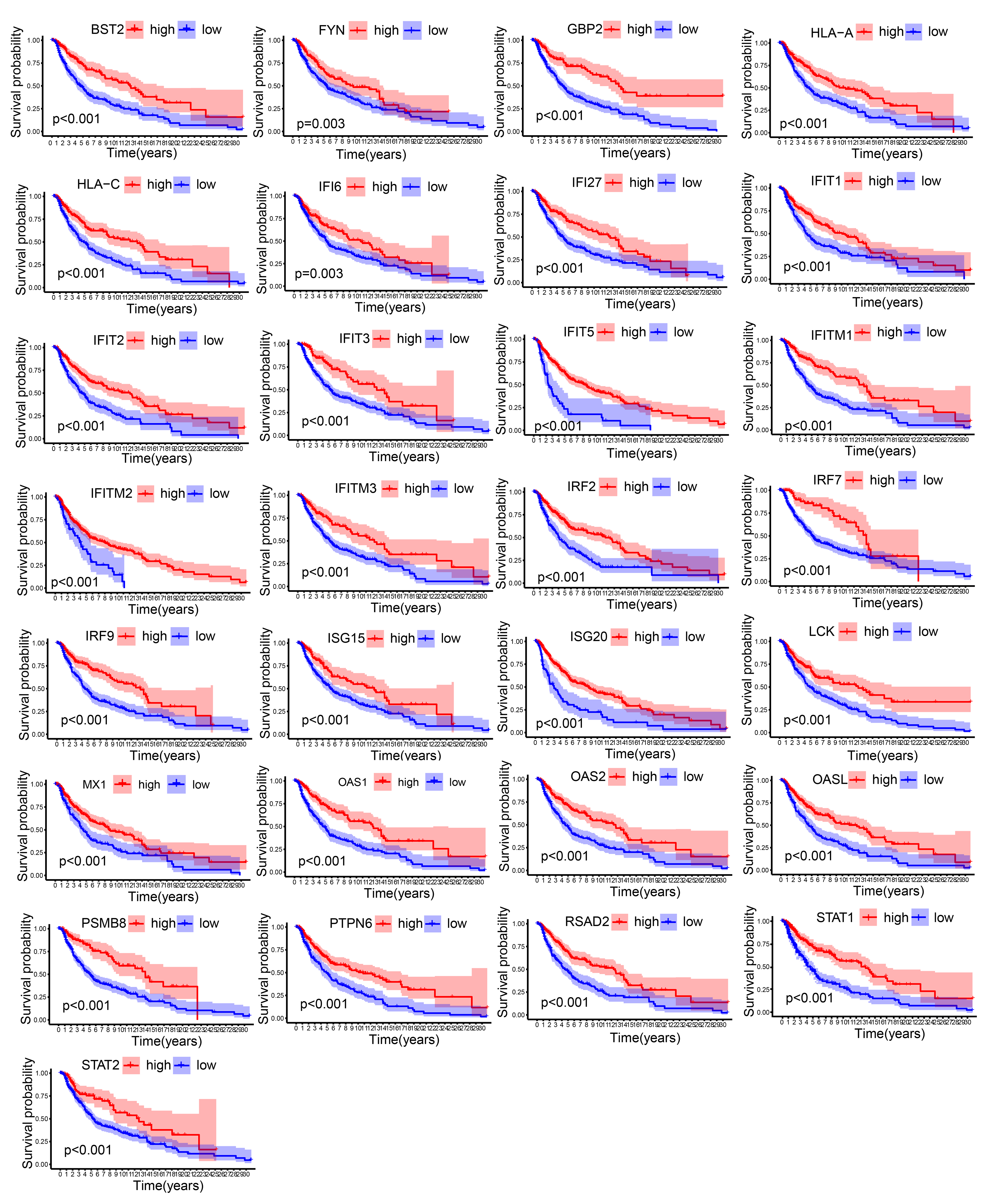

Supplement: Supplementary Figure 1 — Kaplan-Meier curves of 29 hub CDIGs. Kaplan-Meier survival analysis of 29 hub CDIGs in the TCGA cohort. DEGs: differentially expressed genes. [file Image_1.tif]

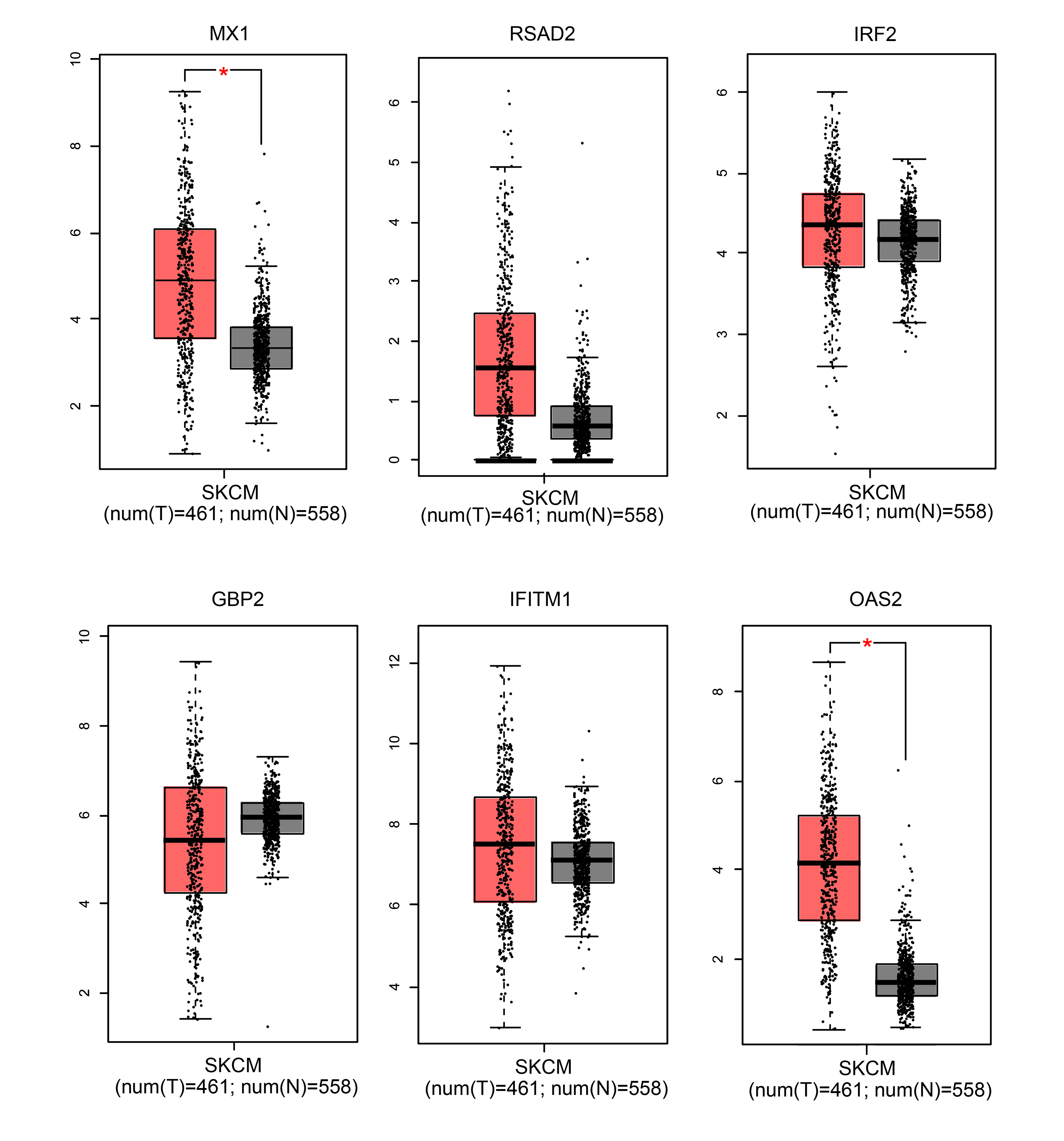

Supplement: Supplementary Figure 2 — The expression levels of CDIGPM genes in SKCM. The expression levels of MX1, RSAD2, IRF2, GBP2, IFITM1, and OAS2 in SKCM were explored using GEPIA. Based on TCGA and GTEx data, the expression levels of MX1 and OAS2 are higher in SKCM cells. CDIGPM: CD8+ T cells-associated immune genes prognostic model. [file Image_2.tif]

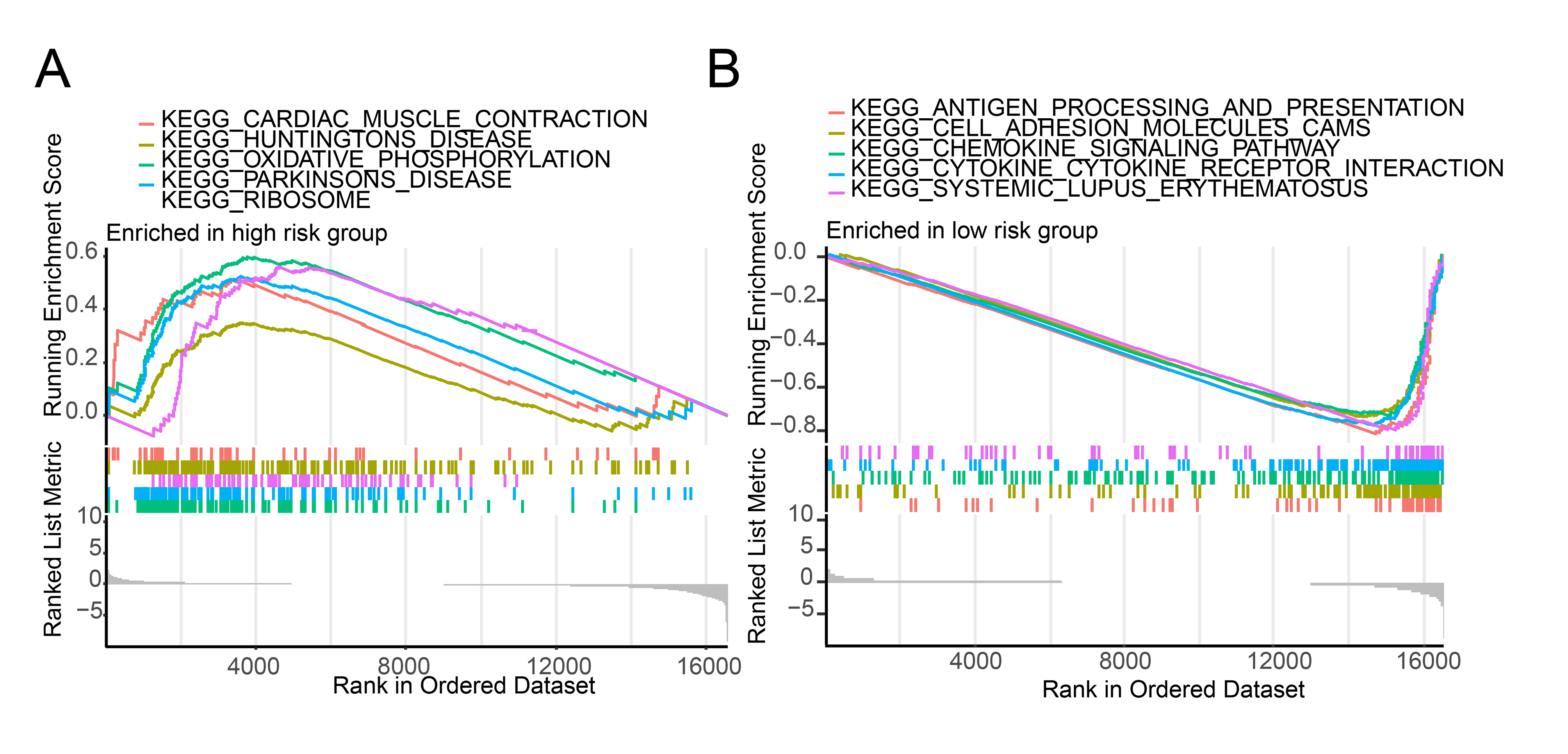

Supplement: Supplementary Figure 3 — Enriched KEGG gene sets in high- and low-CDIGPM groups. (A) KEGG gene sets enriched in high-CDIGPM group. (B) KEGG gene sets enriched in low-CDIGPM group. CDIGPM: CD8+ T cells-associated immune genes prognostic model. [file Image_3.tif]

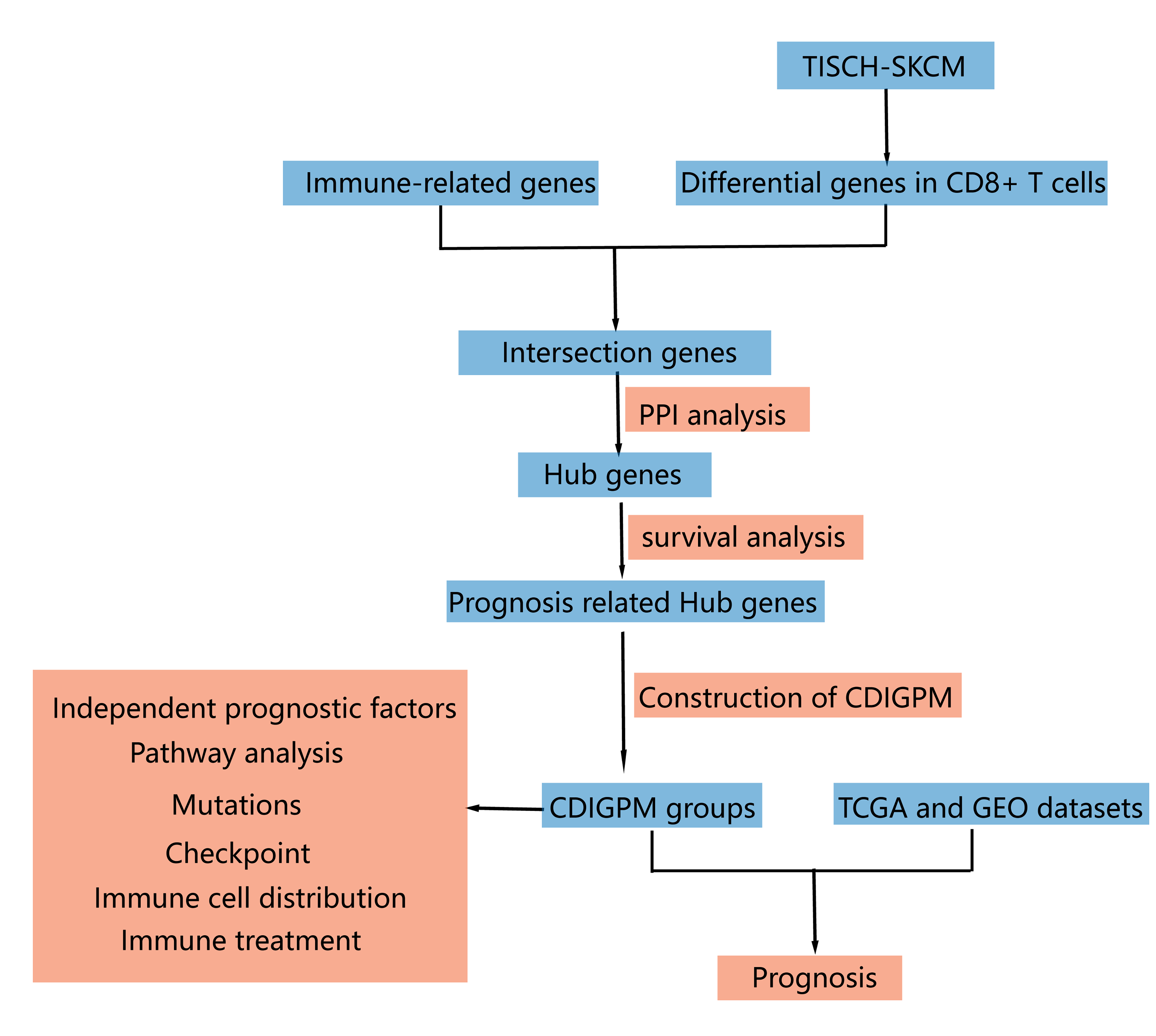

Supplement: Supplementary Figure 4 — Graphical Abstract. [file Image_4.tif]
