# Supplementary material for: A CD8+ T cell-associated immune gene panel for prediction of the prognosis and immunotherapeutic effect of melanoma
Source: Front Immunol. 2022 Oct 20;13:1039565. doi: 10.3389/fimmu.2022.1039565 (PMC9633226; doi:10.3389/fimmu.2022.1039565)
Supplement: Supplementary file 7 [file Table_3.docx]

| **Table S3.** The coefficient of MX1, RSAD2, IRF2, GBP2, IFITM1, and OAS2. | | |
| --- | --- | --- |
| Id | Coefficient |  |
| MX1 | 0.107453066246722 |  |
| RSAD2 | -0.2394476075637 |  |
| IRF2 | -0.419071422539304 |  |
| GBP2 | -0.155211634843746 |  |
| IFITM1 | -0.167386556067903 |  |
| OAS2 | 0.199551721195197 |  |
