# Supplementary material for: A CD8+ T cell-associated immune gene panel for prediction of the prognosis and immunotherapeutic effect of melanoma
Source: Front Immunol. 2022 Oct 20;13:1039565. doi: 10.3389/fimmu.2022.1039565 (PMC9633226; doi:10.3389/fimmu.2022.1039565)
Supplement: Supplementary file 8 [file Table_4.docx]

| **Uni-Cox** | | | | |
| --- | --- | --- | --- | --- |
| ID | HR | HR.95L | HR.95H | pvalue |
| Age | 1.01981584804478 | 1.00986844962803 | 1.02986123024872 | 8.7256188398524e-05 |
| Gender | 0.972812792290296 | 0.721876025168814 | 1.31097958076988 | 0.856303181754546 |
| Stage | 1.37563416543897 | 1.17074264081347 | 1.61638373042269 | 0.000106313041313611 |
| riskScore | 2.04754576019182 | 1.72095397342726 | 2.43611607562654 | 6.29988749122098e-16 |
| **Multi-Cox** | | | | |
| ID | HR | HR.95L | HR.95H | pvalue |
| Age | 1.01413777366134 | 1.00438715437094 | 1.02398305224336 | 0.0043989822592914 |
| Stage | 1.39934361033494 | 1.18235518954326 | 1.65615422260856 | 9.2889224788831e-05 |
| riskScore | 1.9653735326404 | 1.65255925604497 | 2.33740067635934 | 2.18546949438099e-14 |

**Table S4.** Univariate Cox analysis and multivariate Cox analysis of clinical factors and the CDIGPM score.
